# A Neural Network Framework for Predicting the Tissue-of-Origin of 15 Common Cancer Types Based on RNA-Seq Data

**DOI:** 10.3389/fbioe.2020.00737

**Published:** 2020-08-05

**Authors:** Binsheng He, Yanxiang Zhang, Zhen Zhou, Bo Wang, Yuebin Liang, Jidong Lang, Huixin Lin, Pingping Bing, Lan Yu, Dejun Sun, Huaiqing Luo, Jialiang Yang, Geng Tian

**Affiliations:** ^1^Academician Workstation, Changsha Medical University, Changsha, China; ^2^Geneis (Beijing) Co., Ltd., Beijing, China; ^3^Department of Radiology, Beijing Chest Hospital, Capital Medical University, Beijing Tuberculosis and Thoracic Tumor Research Institute, Beijing, China; ^4^Inner Mongolia People’s Hospital, Huhhot, China

**Keywords:** cancer of unknown primary, tissue-of-origin, neural network, RNA sequencing, the Pearson correlation

## Abstract

Sequencing-based identification of tumor tissue-of-origin (TOO) is critical for patients with cancer of unknown primary lesions. Even if the TOO of a tumor can be diagnosed by clinicopathological observation, reevaluations by computational methods can help avoid misdiagnosis. In this study, we developed a neural network (NN) framework using the expression of a 150-gene panel to infer the tumor TOO for 15 common solid tumor cancer types, including lung, breast, liver, colorectal, gastroesophageal, ovarian, cervical, endometrial, pancreatic, bladder, head and neck, thyroid, prostate, kidney, and brain cancers. To begin with, we downloaded the RNA-Seq data of 7,460 primary tumor samples across the above mentioned 15 cancer types, with each type of cancer having between 142 and 1,052 samples, from the cancer genome atlas. Then, we performed feature selection by the Pearson correlation method and performed a 150-gene panel analysis; the genes were significantly enriched in the GO:2001242 Regulation of intrinsic apoptotic signaling pathway and the GO:0009755 Hormone-mediated signaling pathway and other similar functions. Next, we developed a novel NN model using the 150 genes to predict tumor TOO for the 15 cancer types. The average prediction sensitivity and precision of the framework are 93.36 and 94.07%, respectively, for the 7,460 tumor samples based on the 10-fold cross-validation; however, the prediction sensitivity and precision for a few specific cancers, like prostate cancer, reached 100%. We also tested the trained model on a 20-sample independent dataset with metastatic tumor, and achieved an 80% accuracy. In summary, we present here a highly accurate method to infer tumor TOO, which has potential clinical implementation.

## Introduction

Worldwide, almost one in three cancer patients is clinically diagnosed with distant metastases. In most cases, primary and metastatic lesions are identified simultaneously; however, some primary tumors cannot be found after systematic clinicopathological diagnosis (Tomuleasa et al., 2017). Cases with cancer of unknown primary (CUP) lesions account for approximately 3–5% of all newly diagnosed cancers (Richardson et al., 2015); due to its poor prognosis, CUP is the fourth-highest cause of cancer-related deaths around the world (Pavlidis and Fizazi, 2005; Kamposioras et al., 2013). Cancer of unknown primary patients are generally treated with non-selective empirical chemotherapy, which leads to a very low short-term survival rate (Kurahashi et al., 2013). Thus, identifying the primary site is critical for improving long-term survival in CUP patients, especially when considering cancer-type specific targeted therapy (Hudis, 2007; Varadhachary et al., 2008; Hyphantis et al., 2013).

To identify the primary lesion of CUP, a systematic assessment is performed which consists of physical examination, patient-history analysis, serum markers, radiological imaging; as well as immunohistochemical analysis. Immunohistochemical markers are very important for determining tissue-of-origin (TOO; MacReady, 2010; Molina et al., 2012; Oien and Dennis, 2012; Pavlidis and Pentheroudakis, 2012); however, the expressed markers may be non-specific sometimes (Handorf et al., 2013; Montezuma et al., 2013; Tothill et al., 2013). Recently, studies have shown that cellular-origin signatures, which are sufficiently retained in primary tissue, persist after primary cancer cells undergo dedifferentiation and colonization in different tissue types (Ma et al., 2005; Tothill et al., 2005). Molecular profiling is a promising technique that can improve primary-site diagnosis in CUP patients (Ma et al., 2005; Lazaridis et al., 2008; Meiri et al., 2012); it is based on expression microarrays and the quantitative real-time polymerase chain reaction (qRT-PCR) experimental platform (Ma et al., 2005; Lazaridis et al., 2008; Greco et al., 2012; Meiri et al., 2012).

In recent years, cancer classification based on gene expression data such as RT-PCR has attracted great interest and has been implemented in different studies (Lapointe et al., 2004; Mramor et al., 2007; Liu et al., 2008). Single studies are prone to laboratory-specific bias; they are usually limited to a relatively small number of samples and fail to yield novel markers for clinical application. However, applying Next Generation Sequencing (NGS) technology helps alleviate the issue of batch effect by providing gene expression data sets from multiple studies; thus, the integrative analysis of such data can be considered a source of cancer classification. In this regard, establishing a robust classification model is a challenging task; bioinformatics feature selection techniques for establishing such models have been introduced in a previous review (Saeys et al., 2007).

Support vector machines (SVMs) based on the recursive feature elimination (RFE) algorithm represent embedded methods used for feature selection and classification modeling based on microarray gene expression data, which mined 11,925 genes to 154 genes with definite biological significance (Xu et al., 2016). More than 20,000 genes were generated from NGS RNA-Seq data in other studies (Bhowmick et al., 2019); this number is almost twice as much as that from microarray gene expression data. Hence, RNA-Seq data from nine cancer types (lung, liver, colon, thyroid, prostate, bladder, kidney, brain, and skin) were analyzed with different algorithms, and Artificial Bee Colony (ABC) yielded better results than Ant Colony Optimization, Differential Evolution, and Particle Swarm Optimization. Among different cancer types, lower grade brain glioma had the highest accuracy (99.1%) based on the ABC algorithm (Bhowmick et al., 2019). However, the robustness of feature selection and classification modeling methods still needs to be comprehensively evaluated; different algorithms might result in different results depending on their model (Chopra et al., 2010; Bhowmick et al., 2019). Therefore, it is necessary to design a robust classification algorithm based on NGS data that can yield accurate cancer type classification and supplement clinical examination.

In the present study, genome-wide gene expression profiles were established based on comprehensive RNA-Seq data. The gene expression data of ∼8,000 tumor samples were used to identify gene signatures for 15 common human cancer types (lung, breast, liver, colorectal, gastroesophageal, ovarian, cervical, endometrial, pancreatic, bladder, head and neck, thyroid, prostate, kidney, and brain). To screen gene features and evaluate cancer classifiers, the Pearson correlation Neural Network (NN) algorithm was implemented in this study to identify tumor origins.

## Materials and Methods

### RNA-Seq Datasets

NGS-based gene expression profiling data of 7,480 tumor samples were collected from The Cancer Genome Atlas (TCGA, release version v26),^1^ and the tissue origins of those samples were confirmed through histopathological analysis. The downloaded data offered RNA-seq data of 21 cancer types that belongs to projects from United States, which is sequenced using the same protocols. Among them, melanoma had a distinct distribution from other cancer types (80 samples were sampled from primary tumor and 352 were sampled from metastatic tumor) and was excluded. Thus, the expression profiles of 15 common cancer types (lung, breast, liver, colorectal, gastroesophageal, ovarian, cervical, endometrial, pancreatic, bladder, head and neck, thyroid, prostate, kidney, and brain) were studied in this work. The normalized expression value of expression data was downloaded from TCGA and provided the expression levels of 20,501 unique genes for the 15 chosen cancer types.

To perform the bioinformatics analysis in this study, the transcript level of genes was normalized again to form a matrix with rows of sample numbers and columns of gene numbers. The normalization was done by dividing the sum of the gene expression value of each sample. Normalized gene expression data were extracted and represented as a matrix with ‘*m*’ rows and ‘*n*’ columns, such that ‘*m*’ represented 7,480 tumor samples and ‘*n*’ represented the expression levels of 20,501 unique genes.

For log transformation, we used log_2_ to transform the original dataset after replacing zeros to global minimum × 0.1. No normalizations were done after feature selection.

Among all the samples, 7,460 samples were sampled from primary tumors, remaining 20 samples sampled from the metastatic tumors.

### Gene Feature Identification

To identify an optimal gene signature, we introduced a strategy of feature selection and multi-class classification modeling in this study. According to the mechanism of feature selection, the sets of genes were screened by the Pearson Correlation algorithm (Hall, 1998; Saeys et al., 2007). This study consisted of the following steps: (i) create an array to binarize rows for each cancer type (*C* columns) for the *m* tumor samples, labeling the sample as “true” if the sample belongs to the cancer type, otherwise the sample was labeled as “False,” where *C* is the total cancer types and *m* is the sample number; (ii) calculate the correlation of gene expression level with samples labeled “true” for each cancer type, then sort in decreasing order according to their correlation; (iii) take the most important signatures, appeared top *N* of the list, for each cancer type, where *N* is an integer; and (iv) combine *C* lists of the top *N* genes and remove the redundant genes, generating a gene set. Gene expression values from the gene set will be extracted for further usage.

### Feature Performance Assessment

We used a NN (Hinton, 1989) to train the classification model. The gene expression values were used as input signatures for the NN. The NN was designed with three layers, in which the input layer has *N* units, the hidden layer has 50 units, and output layer has 15 units corresponding to each cancer where *N* is the gene number of the input matrix. The output layer of the NN was used as the input for the Softmax function to obtain the probabilities for each cancer type. To prevent overfitting, L2 penalty was set to 0.0001. For comparison, we used logistic regression as a baseline method. The parameter C was set to 10,000 for logistic regression. The algorithms were implemented using scikit-learn package (Pedregosa et al., 2011).

### Gene Ontology Analysis

To perform the Gene ontology (GO) analysis of the identified gene features, GO consortium (Ashburner et al., 2000) was used. The enrichment result was generated by clusterProfiler, which performs a hyper geometric test between the tested genes and gene sets in GO terms (Yu et al., 2012). The biological significances of the selected genes were examined by GO enrichment analysis to identify the most enriched biological-process terms. Benjamini–Hochberg was used to adjust the *p* value.

## Results

### Collection of Gene Expression Datasets of Common Human Cancer Types

**FIGURE 1:**
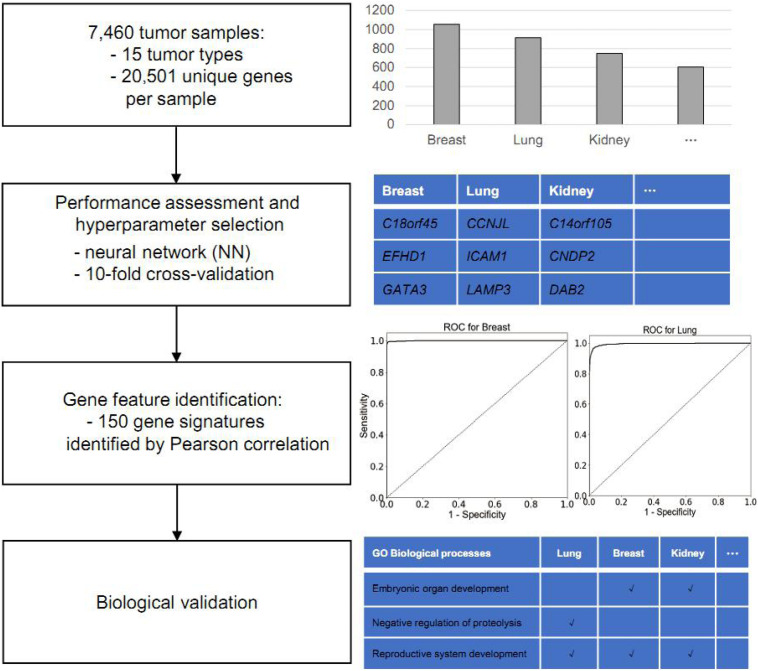
Workflow of gene-feature identification and performance assessment.

The main objective in this study is to identify putative gene biomarkers to classify cancer type. The workflow of the present study is shown in Figure 1. For this analysis, the TCGA was used to obtain gene expression profiles of 15 common solid tumor cancer types via NGS-based RNA-Seq, including lung, gastroesophageal, colorectal, liver, breast, thyroid, cervical, brain, pancreatic, ovarian, endometrial, bladder, kidney, head and neck, and prostate. In total, the expression data of 7,480 tumor samples were collected. Among those, the gene expression profiles of lung adenocarcinoma and lung squamous cell carcinoma samples were merged into lung cancer; those of colon adenocarcinoma and rectum adenocarcinoma were merged into colorectal cancer; those of kidney renal clear cell carcinoma and kidney renal papillary cell carcinoma were merged into kidney cancer; and those of glioblastoma multiforme and lower grade glioma were merged into brain cancer.

Around 20 of the 7,480 samples were sampled from metastatic tumors, whereas 7,460 were sampled from primary tumors. Thus, we split the dataset into the 7,460-sample training dataset and the 20-sample test dataset according to the sampling tumor type. All cancer types in the training dataset had more than 100 samples; the largest sample size was that of breast cancer (1,056 samples), whereas, the smallest sample size was that of pancreatic cancer (142 samples). Table 1 summarizes the datasets and provides information on the tumor samples.

**TABLE 1:** Summary of samples used in the experiments.

**Sampling site**	**Cancer type**	**Code**	**Sample size**	**Percentage (%)**
Primary	Lung	LUAD + LUSC	914	12.25
	Gastroesophageal	STAD	415	5.56
	Colorectal	COAD + READ	604	8.10
	Liver	LIHC	294	3.94
	Breast	BRCA	1056	14.16
	Thyroid	THCA	500	6.70
	Cervical	CESC	258	3.46
	Brain	GBM + LGG	529	7.94
	Pancreatic	PAAD	142	1.90
	Ovary	OV	261	3.50
	Endometrial	UCEC	516	6.92
	Bladder	BLCA	301	4.03
	Kidney	KIRC + KIRP	748	10.03
	Head and Neck	HNSC	480	6.43
	Prostate	PRAD	379	5.08
	Total for primary tumors		7,460	100
Metastatic	Breast	BRCA	7	35.00
	Cervical	CESC	2	10.00
	Colorectal	COAD + READ	1	5.00
	Head and Neck	HNSC	2	10.00
	Thyroid	THCA	8	40.00
	Total for metastatic tumors		20	100

### Hundred and Fifty as a Feature Number Works Well With the Neural Network

A classification modeling database of 15 common cancer types was established based on the expression data of 20,501 unique genes obtained from TCGA. However, having a large number of samples per cancer type might result in variations due to intra-tumor heterogeneity; hence, it is critical to identify the gene expression features from high-dimension datasets. Pearson correlation-based feature selection represents a multivariable filter method for high-dimension data analysis (Hall, 1998; Saeys et al., 2007), which is fast in operation and simple in complex computation; they are used to assess the correlation between cancer type and corresponding gene-expression features. Here, we used Pearson correlation to select the gene-expression signature from NGS-based mRNA expression data for each cancer type. In this study, we used integers from 1 to 20 as candidates for gene number for each cancer type, which might give rise to 20 possible gene sets of 15, 30, …, 300 with a step of 15.

The regression model is an important mathematical model for classification. NNs, as types of deep learning algorithms, are advanced techniques that can analyze complex and high-dimensional data. NNs have been applied in protein classification (Asgari and Mofrad, 2015) and anomaly classification (Suk and Shen, 2013; Plis et al., 2014; Hua et al., 2015). Here, we used NNs as the classification model to assess the performance of different numbers of features. The gene expressions levels were the input layer for the NN; 15 cancer types were the output layer obtained from NNs.

Usually, 10-fold cross-validation is used for minimizing the over-fitting issues and obtaining good performance. Hence, to avoid overfitting of the NN algorithm, we ran a 10-fold cross-validation 10 times using the 7,460-sample training dataset to obtain relatively stable and reliable results, possibly minimizing the percentage of false positives and false negatives. The 10-fold cross validation was performed as follows. (a) Split the whole training dataset into 10 disjoint parts randomly. (b) Use 9 parts as the training set (9/10 training set). (c) Choose *N* genes using Pearson correlation from the 9/10 training set, where *N* is the gene number which might be 15, 30, …, 300 with a step of 15. (d) Train a model using the selected genes using the 9/10 training set. (e) Use the remaining one part as test set as the validation set of the previously trained model. (f) Repeat b–e 10 times with each part being the test set, until all the samples are predicted once. Finally, (g) merge the results from the test parts and evaluate the metrics.

**FIGURE 2:**
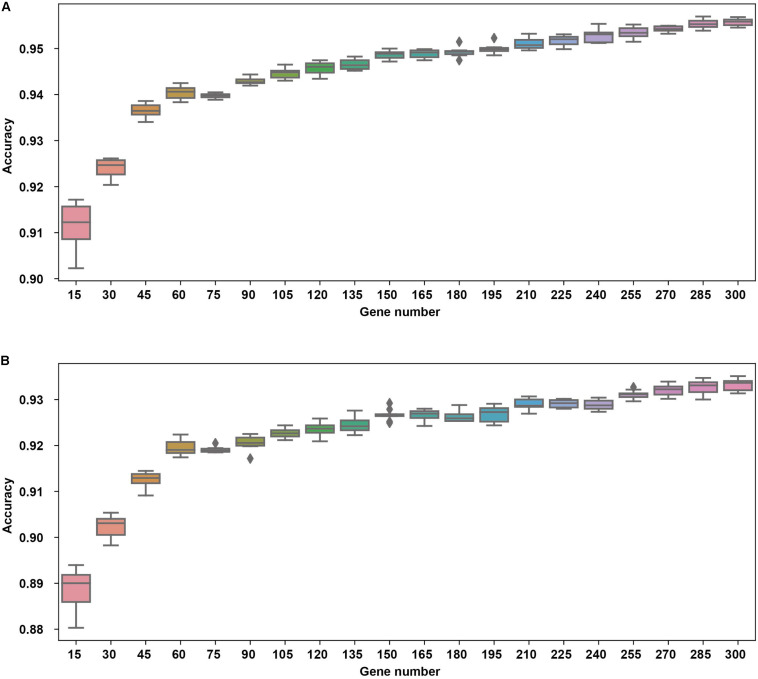
The cross validation accuracy of different gene numbers using neural network **(A)** and logistic regression **(B)**.

**FIGURE 3:**
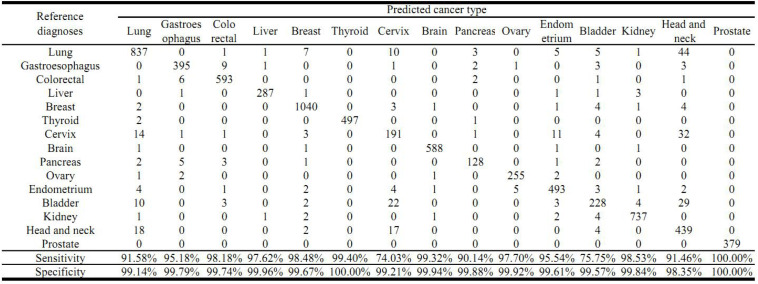
Prediction of cancer type by confusion matrix analysis. The confusion matrix is from one 10-fold cross validation and displayed the relationship between reference diagnosis and the predicted cancer type. The first column represents reference diagnoses; the predicted cancer types by transcript levels of the 150 genes are shown across the top row.

The cross validation was done using different gene number and the accuracies from each 10-fold cross validation are plotted. For comparison, we also used logistic regression as a baseline model (Figure 2). We achieved a good accuracy when the selected gene number is 150. Though a better accuracy could be achieved using the 200 or more as the feature number, the growth curve of number-accuracy is slowing down. The 150 could be seen as a turning point for this curve. Thus, we finally chose the number 150 as the feature number. The results was calculated by averaging the results of 10 times of 10-fold cross validations and showed that the overall accuracy of each cancer type was 94.87% using 150 as the feature number; the sensitivity was on average 93.36%, while the precision was on average 94.07%, corresponding to the actual numbers of cancer samples (Table 2). Among the 15 cancer types, the classifier sensitivity of 13 cancer types (lung, breast, liver, colorectal, gastroesophageal, ovarian, endometrial, pancreatic, head and neck, thyroid, prostate, kidney, and brain) was more than 90%, with that of prostate cancer having the highest sensitivity (100%). On the contrary, the remaining two cancer types had a sensitivity of <90% (74.75% for bladder cancer and 71.63% for cervical cancer) (Figure 3 and Table 2).

**TABLE 2:** Sensitivity and precision assessment for each cancer type.

	**Sensitivity (%)**	**Precision (%)**
Lung	91.87	92.76
Gastroesophageal	94.89	96.33
Colorectal	98.06	96.88
Liver	97.99	98.80
Breast	98.43	97.98
Thyroid	99.38	99.58
Cervical	71.63	76.38
Brain	99.32	99.41
Pancreatic	91.76	94.63
Ovarian	97.55	97.15
Endometrial	95.54	94.85
Bladder	74.75	88.36
Kidney	98.42	98.54
Head and Neck	90.83	79.39
Prostate	100.00	100.00
Average	93.36	94.07

We also attempted to use the log-transformed data for in the cross validation since log-transformation was a common transformation for gene expression profile. For a reasonable comparison, we selected 10 genes for each cancer in each fold of cross validation. However, the overall accuracy by 10 times of 10-fold cross validations only reached 80.90% (Supplementary Table S1), which is not satisfactory. In contrast, the data by the previously described transformation method output the result of 94.87%, showing more optimization shall be done for a better result using the log-transformed data.

### The Identified Genes Were Enriched in Several Organ-Specific Pathways

**FIGURE 4:**
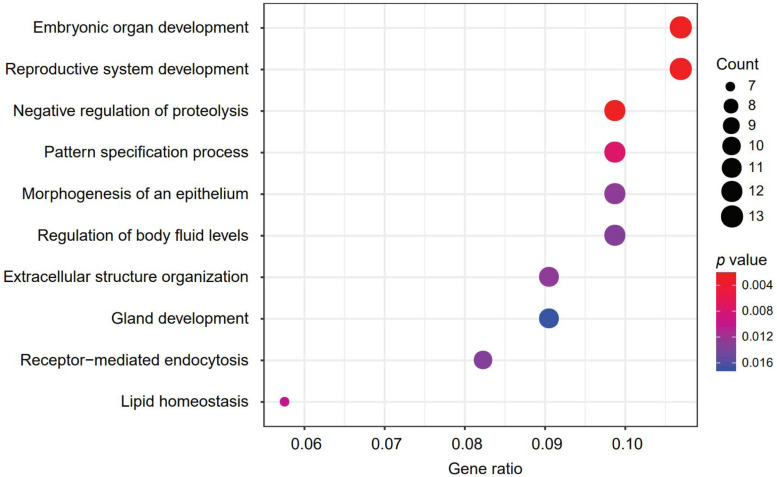
The most represented biological processes associated with our signature genes. Dot plot displaying the number of signature genes involved in each biological process, determined by enrichment analysis. Dot size represents the number of genes, and dot color represents *p*-value; a lower *p*-value represents a higher probability of a biological process being enriched with the signature genes.

A 150-gene set was identified using the whole training dataset for subsequent processing (Table 3). To understand how frequently those genes will show up in the cross validation phase, we counted the genes in all the 100 gene sets used in the cross validation and found that 117 genes out of the 150 gene showed up in all gene sets validation, showing the robustness of the feature selection method based on Pearson correlation (Supplementary Table S2). To investigate the biological processes of the involved signature genes, GO enrichment analysis was performed. We saw that the most functionally enriched processes related to our 150-gene panel by GO analysis were biological processes (Figure 4 and Table 4). Among those, GO:0048568 Embryonic organ development, GO:0061458 Reproductive system development, GO:0007389 Pattern specification process, GO:0043062 Extracellular structure organization, GO:0002009 Morphogenesis of an epithelium, and GO:0048732 Gland development were related to tissue or organ morphogenesis. Our signature genes were involved in these biological processes and might be useful for classifying distinct cancer types. Hence, the enrichment analysis in the present study might provide a basis to improve our understanding of lung, gastroesophageal, colorectal, liver, breast, thyroid, cervical, brain, pancreatic, ovarian, endometrial, bladder, kidney, head and neck, and prostate cancers.

**TABLE 3:** Gene signatures, as identified by Pearson correlation.

**Rank**	**Lung**	**Gastro-esophageal**	**Colo-rectal**	**Liver**	**Breast**	**Ovary**	**Cervix**	**Endometrial**	**Pancreatic**	**Bladder**	**Head and Neck**	**Thyroid**	**Prostate**	**Kidney**	**Brain**
1	*CCNJL*	*BRI3BP*	*C2orf89*	*AMBP*	*C18orf45*	*BCAM*	*C1orf14*	*ASRGL1*	*CASR*	*C10orf116*	*BNC1*	*APLP2*	*C17orf93*	*C14orf105*	*BAALC*
2	*ICAM1*	*CCDC109A*	*CDH17*	*APOB*	*EFHD1*	*C10orf41*	*C9orf53*	*DLX5*	*CTRB1*	*FER1L4*	*CSDAP1*	*CTSB*	*HOXB13*	*CNDP2*	*CACNG7*
3	*LAMP3*	*CDC42EP1*	*CDX1*	*APOC2*	*GATA3*	*GPR27*	*CENPW*	*DLX6*	*CTRB2*	*GRHL3*	*GJB5*	*DAPK2*	*KLK2*	*DAB2*	*CTNND2*
4	*LPCAT1*	*GATA6*	*CDX2*	*ASGR1*	*IRX5*	*HOXD4*	*LOC642587*	*FLJ39739*	*CUZD1*	*KRT7*	*KRT14*	*HHEX*	*KLK3*	*FBXO17*	*FEZ1*
5	*NAPSA*	*GNL3L*	*EPS8L3*	*ASGR2*	*LMX1B*	*KCNK15*	*PSMC3IP*	*LOC442459*	*FFAR1*	*LOC100188947*	*KRT5*	*LCN12*	*KLK4*	*GALNT14*	*GPM6B*
6	*ROS1*	*HIATL2*	*GPA33*	*F2*	*PRLR*	*KLHL14*	*RASIP1*	*LOC643387*	*FOXL1*	*PLA2G2F*	*KRT6A*	*MUC15*	*NKX3-1*	*PKD2*	*LRRC4B*
7	*SFTA2*	*PIAS1*	*GPR35*	*ITIH1*	*TBC1D9*	*WIT1*	*SERPINB3*	*LYPLA2P1*	*INS*	*PPARG*	*KRT6B*	*NKX2-1*	*SLC45A3*	*SLC22A2*	*MGC42105*
8	*SFTPA1*	*POU2F1*	*HEPH*	*PROC*	*TFAP2A*	*WT1*	*SERPINB4*	*MSX1*	*PNLIPRP1*	*SNCG*	*PKP1*	*NPC2*	*STEAP2*	*SLC28A1*	*PPP2R2B*
9	*SFTPA2*	*ZBTB7A*	*NOX1*	*SERPINA10*	*TRPS1*	*ZFP92*	*SMC1B*	*SOX17*	*PRSS3*	*UPK1A*	*PTTG3P*	*TSHR*	*TMPRSS2*	*SLC3A1*	*REEP2*
10	*SFTPB*	*ZFPM1*	*VIL1*	*VTN*	*XBP1*	*ZNF503*	*TCAM1P*	*STX18*	*TRY6*	*UPK2*	*SFN*	*ZBED2*	*TRPV6*	*TMEM140*	*SYT11*

**TABLE 4:** The overrepresented biological processes associated with identified gene-signatures, as obtained through GO enrichment analysis.

**GO biological processes**	**Lung**	**Gastro- esophageal**	**Colo-rectal**	**Liver**	**Breast**	**Thyroid**	**Cervical**	**Brain**	**Pancreatic**	**Ovarian**	**Endo- metrial**	**Bladder**	**Kidney**	**Head and neck**	**Prostate**
GO:0048568 Embryonic organ development		√	√		√					√	√	√	√	√	
GO:0045861 Negative regulation of proteolysis	√		√	√		√	√		√					√	
GO:0061458 Reproductive system development	√	√	√		√	√				√		√	√	√	√
GO:0007389 Pattern specification process			√		√	√				√	√	√	√		√
GO:0055088 Lipid homeostasis				√	√	√			√			√			
GO:0043062 Extracellular structure organization	√		√	√				√	√	√					√
GO:0002009 Morphogenesis of an epithelium					√	√	√		√	√	√	√	√		√
GO:0006898 Receptor-mediated endocytosis				√				√					√		√
GO:0050878 Regulation of body fluid levels		√		√	√							√	√	√	
GO:0048732 Gland development		√		√	√	√				√	√		√		√
GO:2001242 Regulation of intrinsic apoptotic signaling pathway			√		√	√			√		√				√
GO:0009755 Hormone-mediated signaling pathway		√			√	√						√	√		√

### The Trained Neural Network Showed High Accuracy on Independent Metastatic Tumor Dataset

We further sought to validate our model on the 20-sample metastatic dataset as a test set. We trained the NN model and the logistic regression model on the whole training dataset using the 150-gene set, which was then used for predicting the test set. The prediction accuracy of NNs was 80%, while the prediction accuracy of the logistic regression model was 70%. The detailed predictions are shown in Table 5.

**TABLE 5:** The performance on metastatic samples of the neural network trained on the primary samples.

**Sample Id**	**predicted_ by_NN**	**predicted_by _logistic**	**true_label**
TCGA-AC-A6IX-06A-11R-A32P-07	BRCA	BRCA	BRCA
TCGA-BH-A18V-06A-11R-A213-07	BRCA	BLCA	BRCA
TCGA-BH-A1ES-06A-12R-A24H-07	BRCA	LIHC	BRCA
TCGA-BH-A1FE-06A-11R-A213-07	KIDNEY	KIDNEY	BRCA
TCGA-E2-A15A-06A-11R-A12D-07	BRCA	BRCA	BRCA
TCGA-E2-A15E-06A-11R-A12D-07	BRCA	BRCA	BRCA
TCGA-E2-A15K-06A-11R-A12P-07	BRCA	BRCA	BRCA
TCGA-HM-A6W2-06A-22R-A33Z-07	UCEC	UCEC	CESC
TCGA-UC-A7PG-06A-11R-A42S-07	CESC	CESC	CESC
TCGA-NH-A8F7-06A-31R-A41B-07	COAD + READ	COADREAD	COAD + READ
TCGA-KU-A6H7-06A-21R-A31N-07	CESC	CESC	HNSC
TCGA-UF-A71A-06A-11R-A39I-07	LUNG	LUNG	HNSC
TCGA-DE-A4MD-06A-11R-A250-07	THCA	THCA	THCA
TCGA-EM-A2CS-06A-11R-A180-07	THCA	THCA	THCA
TCGA-EM-A2P1-06A-11R-A206-07	THCA	THCA	THCA
TCGA-EM-A3FQ-06A-11R-A21D-07	THCA	THCA	THCA
TCGA-EM-A3SU-06A-11R-A22U-07	THCA	THCA	THCA
TCGA-J8-A3O2-06A-11R-A23N-07	THCA	THCA	THCA
TCGA-J8-A3YH-06A-11R-A23N-07	THCA	THCA	THCA
TCGA-J8-A4HW-06A-11R-A250-07	THCA	THCA	THCA

## Discussion

Inferring cancer TOO is important for CUP patients and might serve well for minimizing misdiagnosis, even if the cancer origin is diagnosed by pathological observation. Hence, it is critical to develop a method to classify TOO of common cancer types. This study was possible because of the great advancements in NGS technologies and the general application of NGS in clinical experiments, along with the efforts made by researchers who have contributed to the TCGA, from where huge gene expression datasets can be obtained. In the present study, we utilized the NN method to comprehensively analyze high-dimensional RNA-Seq datasets of 15 common cancer types. The 150-gene panel of cancer classifiers demonstrated an average accuracy of 94.87%, corresponding to the actual numbers of cancer samples.

Several hallmarked studies indicated that the cellular origin signatures that are expressed in primary tissue are sufficiently retained even after primary cancer cells undergo dedifferentiation and colonization in different tissue types (Ma et al., 2005; Tothill et al., 2005). A recent study compared four different algorithms and indicated that the modeling performance differed between these algorithms when analyzing RNA-Seq data from 4,127 primary tumor tissue samples related to nine cancer types (Bhowmick et al., 2019). Among those, ABC yielded the best results; it had an average precision of 91.16% and an average sensitivity of 96.5% for nine cancer types (Bhowmick et al., 2019). However, our study demonstrated an average precision of 94.07% and an average sensitivity of 93.36%, corresponding to 7,460 cancer samples related to 15 common cancer types. Although the average sensitivity from our study was a bit lower than that of ABC algorithm, we managed to dramatically minimize the false-positive rate to 0.34% (Table 2). Moreover, the overall accuracy with an average of 94.87% is higher than that of other gene expression-based signatures, which ranged from 79–91% (Ma et al., 2005; Monzon et al., 2009; Kerr et al., 2012). Furthermore, the performance of the 150-gene panel was higher than that of the immunohistochemistry technique (75%), which represents the current clinical practice standard, as tested by a 10-antibody panel (Park et al., 2007).

In the present study, GO analysis revealed several over-represented biological processes related to tissue morphogenesis, such as embryonic organ development, reproductive system development, pattern specification process/regionalization, extracellular structure organization, epithelial morphogenesis, and glandular development (Figure 4 and Table 4). Additionally, the expression patterns of several signature genes of the 150-gene panel were previously reported to be related to tissues of specific tumor types. For example, *GRHL3* (*Grainyhead-Like Transcription Factor 3*) encodes a cancer suppressor that is a member of the grainyhead-like transcription factor family (Darido et al., 2011). The downregulated *GRHL3* gene was associated with head and neck squamous cell carcinomas (Frisch et al., 2018); overexpression of the oncogenic mir21 was as result of decreased GRHL3 (Bhandari et al., 2013). In addition, *KLKs* (*Kallikrein-Related Peptidases*) are genes that encode serine proteases that exhibit a deregulated expression in prostate cancer. In our study, *KLK2, KLK3*, and *KLK4* were identified as gene signatures for prostate cancer; KLK3 is a prostate-specific antigen that is a gold-standard clinical biomarker widely employed in the diagnosis and monitoring of prostate cancer (Fuhrman-Luck et al., 2014); KLK2 showed promise as prostate cancer biomarker, as well. Additionally, the deregulated expression of KLKs has been utilized in designing novel therapeutic targets for prostate cancer (Fuhrman-Luck et al., 2014).

GATA DNA-binding proteins, commonly abbreviated as GATAs, are zinc-finger binding transcription factors that regulate tissue differentiation and specification (Chou et al., 2010; Zheng and Blobel, 2010). In our study, GATA3 and GATA6 transcripts were identified as gene signatures for breast cancer and gastroesophageal cancer, respectively. Previous studies have indicated that GATA3 was weakly expressed in a wide variety of normal tissues, while its expression was remarkably elevated in breast cancer (Yang and Nonaka, 2010; Liu et al., 2012); moreover, GATA3 has been identified as a novel clinical marker for detecting primary and metastatic breast cancer (Cimino-Mathews et al., 2013; Krings et al., 2014; Shield et al., 2014; Braxton et al., 2015; Sangoi et al., 2016; Yang et al., 2017). GATA6 was initially cloned from rat gastric tissue, designated as GATA-GT1 (Tamura et al., 1994); however, recent studies have indicated that GATA6 was frequently overexpressed and/or amplified in human gastroesophageal cancer (Sulahian et al., 2014; Chia et al., 2015; Song et al., 2018). There’s some limitations about our studies. First, we assessed the model based on NGS RNA-Seq data from the formalin-fixed and paraffin-embedded materials, but not fresh materials. We did not evaluate it in fresh materials mainly due to the formalin-fixed and paraffin-embedded materials are most diagnostic materials in routine practice. Second, some solid tumor cancer types such as sarcoma was not included due to the unavailability of RNAseq data; besides, the non-solid tumors were currently excluded; melanoma was also excluded due to the data scarcity and the distinct distribution of its primary tumor sample number and metastatic tumor sample number. Thus, further efforts should be made for a broader application scope. Third, the training dataset could be further expanded. Since the final gene set contains some organ development-related genes, we can infer that the gene set does not only classify cancer types, but also organs. Staub et al. has already made efforts by expand the training dataset and achieved a better result (Staub et al., 2009). Thus, expression profiles from normal tissues could be further added to our training dataset for a better performance. Another limitation is that our method is based on the expression value without any manipulations. Recently, an algorithm called TSP was applied to this problem, which will generate gene pairs instead of single gene features, giving rise to a leap to the prediction accuracy (Shen et al., 2020). We believe that combining the neural network and the feature generation could further improve the performance for CUP problems.

## Conclusion

In the present study, our 150-gene panel exhibited promising results as a tumor classifier for inferring the origin of tumor tissue. First, we obtained NGS-based RNA-Seq data for 7,460 tumor samples from TCGA. Second, we built a fine pipeline to identify gene signatures based on their transcript-levels for 15 common cancer types. Third, we utilized the Neural Network to evaluate the performance of the genes; on average, the precision was 94.07%, while the sensitivity was 93.36%. In addition, GO enrichment analysis revealed several biological processes, including tissue morphogenesis; notably, most of the gene signatures were involved in key oncogenic pathways, supporting our 150-gene panel. Therefore, the 150-gene biomarker signature in our study might prove to be clinically useful for identifying cancers of unknown origin and confirming initial clinical diagnoses. In future studies, we will focus on the application of this model in metastatic cancer patients, in addition to patients with cancer of unknown origin, to evaluate their therapy outcome.

## Data Availability Statement

Publicly available datasets were analyzed in this study. This data can be found here: https://dcc.icgc.org/releases/release_26.

## Author Contributions

GT, JY, and HL conceived the concept of the work. BH, BW, YL, and JL performed the experiments. YZ wrote the manuscript. ZZ, HL, PB, LY, and DS reviewed the manuscript. All authors approved the final version of this manuscript.

## Conflict of Interest

YZ, BW, YL, JL, HL, JY, and GT were employed by the company Geneis (Beijing) Co., Ltd. The remaining authors declare that the research was conducted in the absence of any commercial or financial relationships that could be construed as a potential conflict of interest.

## Footnotes

1 https://dcc.icgc.org/releases/release_26

## Supplementary Material

TABLE S1 The result of 10 times of 10-fold cross validations using 10 genes for each cancer.

TABLE S2 The gene sets from the cross validation phase and the occurrence in the final gene set.
